# Angiopoietin-Like Protein 3 (ANGPTL3) Modulates Lipoprotein Metabolism and Dyslipidemia

**DOI:** 10.3390/ijms22147310

**Published:** 2021-07-07

**Authors:** Pei-Yi Chen, Wan-Yun Gao, Je-Wen Liou, Ching-Yen Lin, Ming-Jiuan Wu, Jui-Hung Yen

**Affiliations:** 1 Center of Medical Genetics, Hualien Tzu Chi Hospital, Buddhist Tzu Chi Medical Foundation, Hualien 970, Taiwan; pyc571@gmail.com; 2 Department of Molecular Biology and Human Genetics, Tzu Chi University, Hualien 970, Taiwan; jouyuan22@gmail.com; 3 Institute of Medical Sciences, Tzu Chi University, Hualien 970, Taiwan; 102712131@gms.tcu.edu.tw; 4 Department of Biochemistry, School of Medicine, Tzu Chi University, Hualien 970, Taiwan; jwliou@mail.tcu.edu.tw; 5 Department of Biotechnology, Chia Nan University of Pharmacy and Science, Tainan 717, Taiwan; mingjiuanwu@gmail.com

**Keywords:** dyslipidemia, TG-rich lipoproteins, ASCVDs, ANGPTL3, lipoprotein lipase

## Abstract

Dyslipidemia is characterized by increasing plasma levels of low-density lipoprotein-cholesterol (LDL-C), triglycerides (TGs) and TG-rich lipoproteins (TGRLs) and is a major risk factor for the development of atherosclerotic cardiovascular disorders (ASCVDs). It is important to understand the metabolic mechanisms underlying dyslipidemia to develop effective strategies against ASCVDs. Angiopoietin-like 3 (ANGPTL3), a member of the angiopoietin-like protein family exclusively synthesized in the liver, has been demonstrated to be a critical regulator of lipoprotein metabolism to inhibit lipoprotein lipase (LPL) activity. Genetic, biochemical, and clinical studies in animals and humans have shown that loss of function, inactivation, or downregulated expression of ANGPTL3 is associated with an obvious reduction in plasma levels of TGs, LDL-C, and high-density lipoprotein-cholesterol (HDL-C), atherosclerotic lesions, and the risk of cardiovascular events. Therefore, ANGPTL3 is considered an alternative target for lipid-lowering therapy. Emerging studies have focused on ANGPTL3 inhibition via antisense oligonucleotides (ASOs) and monoclonal antibody-based therapies, which have been carried out in mouse or monkey models and in human clinical studies for the management of dyslipidemia and ASCVDs. This review will summarize the current literature on the important role of ANGPTL3 in controlling lipoprotein metabolism and dyslipidemia, with an emphasis on anti-ANGPTL3 therapies as a potential strategy for the treatment of dyslipidemia and ASCVDs.

## 1. Introduction

**Figure 1:**
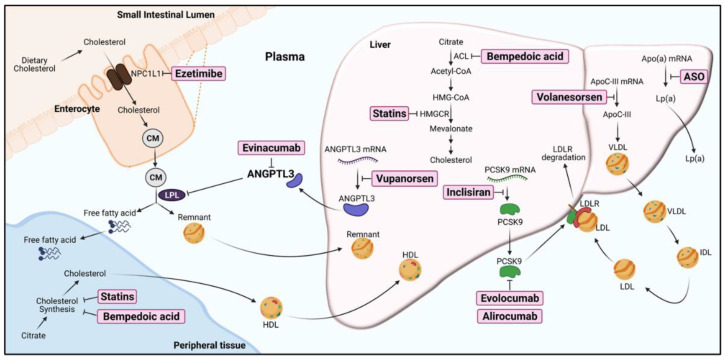
The pharmacological targets of current or potential lipid-lowering agents for dyslipidemia therapy. ACL, ATP-citrate lyase; ANGPTL3, angiopoietin-like protein 3, Apo(a), Apolipoprotein(a); ApoC-III, Apolipoprotein C-III; ASO, antisense oligonucleotides; CM, chylomicrons; HDL, high-density lipoproteins; HMG-CoA, 3-hydroxy-3-methyl-glutaryl-coenzyme A; HMGCR, 3-hydroxy-3-methyl-glutaryl-coenzyme A reductase; IDL, intermediate-density lipoprotein; LDL, low-density lipoproteins; LDLR, low-density lipoprotein receptor; LPL, lipoprotein lipase; Lp(a), lipoprotein (a); NPC1L1, Niemann-Pick C1-like 1 protein; PCSK9, proprotein convertase subtilisin/kexin type 9; VLDL, very-low-density lipoproteins. The approaches for inhibition of targets in lipid/lipoprotein metabolism are highlighted in the boxes (created with BioRender.com, accessed on 2 July 2021).

Dyslipidemia is often characterized by increases in low-density lipoprotein-cholesterol (LDL-C), triglycerides (TGs), and TG-rich lipoproteins (TGRLs) levels in circulation and is associated with obesity, type 2 diabetes, and metabolic syndrome. This condition is a major risk factor for the development of atherosclerotic cardiovascular diseases (ASCVDs) and myocardial infarction (MI) [1,2]. Although the pharmacological reduction of LDL-C by statins, ezetimibe, or PCSK9 inhibitors could effectively treat ASCVDs, a notable residual risk remains, and these disorders are still the leading cause of death in industrialized societies [3,4,5]. In addition, the efficacy of therapeutic medication for LDL-C-lowering agents is poor in patients with familial hypercholesterolemia-related mutations affecting functional LDL receptors. Therefore, in addition to reducing LDL-C levels, exploring novel molecular targets of dyslipidemia therapy is ongoing (Figure 1). Recently, new pharmacological approaches for CVD treatment have focused on the regulation of plasma TGRLs, which carry dietary and synthesized TGs, cholesterol, and cholesterol ester in blood vessels [1,6].

TGRLs are envisioned to play essential roles on the progression of ASCVDs [6]. Variants of gene mutations in TGRL metabolism with high TG concentration in plasma exhibits strong correlation with magnitude of ASCVD risk [7]. TGs are present in the core of TGRLs, which encompass a mixture of chylomicrons and very-low-density lipoprotein (VLDL) particles, and their lipolytic remnants. The TGs could be hydrolyzed to free fatty acids by lipoprotein lipase (LPL), which is anchored on the luminal surface of capillaries [8]. When LPL-mediated TGs hydrolysis is impeded, accumulation of substrate TGRL particles in plasma occurs. Regulation of LPL activity represents an ideal strategy to prevent or treat hypertriglyceridemia and ASCVDs [9]. Recently, angiopoietin-like 3 (ANGPTL3), a member of the angiopoietin-like protein (ANGPTL) family, is shown to inhibit LPL activity and be involved in the regulation of lipoprotein metabolism. Thus, ANGPTL3 is considered a promising pharmacological target for treatment of dyslipidemia [10,11]. In this article, we review the current understanding of ANGPTL3, focusing on its role in the modulation of lipoprotein metabolism, dyslipidemia, and cardiovascular events, and discuss its application as a therapeutic target for the treatment of dyslipidemia and ASCVDs.

## 2. Molecular Features of ANGPTL3

Angiopoietin-like proteins (ANGPTLs) are a family of secreted glycoproteins comprising eight members (ANGPTL1–8). ANGPTLs possess high homology to angiopoietins, which are critical regulators of angiogenesis. However, ANGPTLs could not interact with the angiopoietin receptors Tie1 and Tie2 on the endothelium [12]. Among these ANGPTL members, ANGPTL3, ANGPTL4 and ANGPTL8 share high sequence homology and are involved in the regulation of LPL activity and TGRL metabolism. Current studies in murine models have suggested that ANGPTL3 is a better pharmacological target than ANGPTL4 for the treatment of dyslipidemia and atherosclerosis [13,14,15]. ANGPTL8 acts in concert with ANGPTL3 to inhibit LPL activity and reduce hydrolysis of TGs in capillaries of muscles and adipose tissue [16].

### 2.1. Discovery of ANGPTL3

In 1999, based on the EST databases, Conklin et al. cloned and identified an angiopoietin-like gene, ANGPTL3 [17]. The effect of ANGPTL3 on lipid metabolism was identified by studying the mutations associated with the hypolipidemic phenotype in KK/San mice (a sub-strain of KK obese mice) [18]. Compared with wild-type KK mice, KK/San mice possess a 4-bp insertion in exon 6 of the *ANGPTL3* gene, which introduces a premature stop codon, given the low mRNA levels of ANGPTL3 in these mice. Both overexpression through adenovirus-mediated ANGPTL3 gene transfer and injection of the recombinant ANGPTL3 protein in KK/San or C57BL/6J mice could raise the plasma levels of total cholesterol, TGs, and non-esterified fatty acids (NEFA) in these mice [18]. These data indicated that ANGPTL3 is responsible for hypolipidemia and could regulate lipid metabolism in vivo.

### 2.2. The Structural Features of ANGPTL3

**Figure 2:**
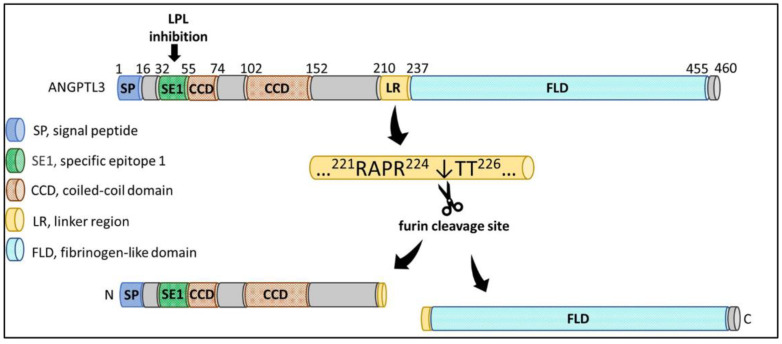
The structural features of ANGPTL3 protein. ANGPTL3 is composed of a signal peptide (SP), an N-terminal coiled-coil domain (CCD) involved in a specific epitope 1 (SE1) for LPL binding and inhibition, a linker region (LR), and a C-terminal fibrinogen-like domain (FLD) with angiogenic properties. The amino acid residues ^221^RAPR^224^↓TT^226^ within the LR could be cleaved by furin to yield a N-terminal CCD and a C-terminal FLD in hepatic cells (created with BioRender.com, accessed on 2 July 2021).

ANGPTL3 is a 70 kDa glycoprotein principally expressed and secreted by hepatocytes. The protein structure of ANGPTL3 is comprised of a 460-amino-acid polypeptide with the characteristic structure of angiopoietins, such as a signal peptide with 16 amino acid residues, an N-terminal coiled-coil domain, a linker region, and a fibrinogen-like C-terminal domain [19,20] (Figure 2). ANGPTL3 can be cleaved at amino acid residues ^221^RAPR^224^↓TT^226^ within the linker region to yield an N-terminal coiled-coil region and a C-terminal fibrinogen-like domain by the proprotein convertase furin (also known as PCSK3) in hepatocytes and by the PACE4 protein (also known as PCSK6) through extracellular cleavage [19,21]. The cleaved N-terminal region of ANGPTL3 could increase plasma TG levels in animal studies and has been found to interact with LPL and endothelial lipase (EL) to inhibit their catalytic activities and TG lipolysis [22,23,24], suggesting that the coiled-coil domain of ANGPTL3 is critical for its activation in vivo. Both a full-length protein and a cleaved N-terminal form of ANGPTL3 were found to circulate in plasma. This truncated ANGPTL3 was found to increase the inhibitory effect on EL but not LPL, indicating that furin-mediated cleavage of ANGPTL3 is more important for EL inhibition [25].

In addition to protein cleavage, ANGPTL3 undergoes O-linked glycosylation by liver-expressed N-acetylgalactosaminyltransferase 2 (GalNAc-T2), encoded by *GALNT2* [26]. The glycosylation at the threonine 226 residue (T226) by GalNAc-T2 is adjacent to the cleavage site of proprotein convertase in ANGPTL3, and this glycosylation hinders protein processing at arginine (R224) [27,28]. These findings suggest that GalNAc-T2-mediated O-glycosylation could modulate ANGPTL3 activation and plasma TG levels.

### 2.3. The Expression of ANGPTL3

The human *ANGPTL3* gene is located on chromosome 1p31.1 and is expressed mainly in the liver at the early stage during liver development, and its expression is maintained in adults [17]. The expression of *ANGPTL3* is mainly controlled by the transcription factors liver X receptor-α (LXRα) and hepatocyte nuclear factor-1α (HNF-1α). The *ANGPTL3* promoter contains an LXR response element (LXRE). The synthetic LXR agonist T0901317 could increase promoter activity and augment ANGPTL3 mRNA and protein expression in hepatic cells [29,30,31]. In animal studies, treatment of mice with T0901317 promoted TGs accumulation in the liver and plasma, which was accompanied by increases in hepatic lipogenic gene and ANGPTL3 expression. However, in *ANGPTL3*-null mice, an LXR agonist could not induce hypertriglyceridemia but did induce the accumulation of TGs in the liver [30]. These findings suggest that the *ANGPTL3* gene is a direct target of LXRα and that hypertriglyceridemia associated with LXR activation is due to overexpression of hepatic ANGPTL3.

Fugier et al. reported that ANGPTL3 mRNA was markedly reduced by approximately 70% in a thyroid hormone receptor (TRβ)-dependent manner after a subcutaneous injection of thyroid hormone (T3) in hypothyroid rats. In contrast, this inhibitory effect of T3 on ANGPTL3 mRNA expression was not observed in TRβ-deficient animals. Mutation of the HNF-1α binding site within the *ANGPTL3* promoter completely abolished TRβ-mediated promoter repression [32]. However, TRβ antagonized HNF-1α without interrupting its DNA-binding capacity, suggesting that TRβ can sequester a coactivator recruited by HNF-1α upon the *ANGPTL3* promoter. Additionally, several studies have found that other factors, such as insulin, leptin, and statins, could suppress ANGPTL3 expression in mice or hepatic cell lines [33,34,35,36]. In addition to lipid metabolism, these findings suggest that ANGPTL3 may also be involved in glucose metabolism in diabetic patients.

## 3. ANGPTL3 and Lipid Metabolism

### 3.1. The Function of ANGPTL3 in Lipoprotein Lipase (LPL) Inhibition

**Figure 3:**
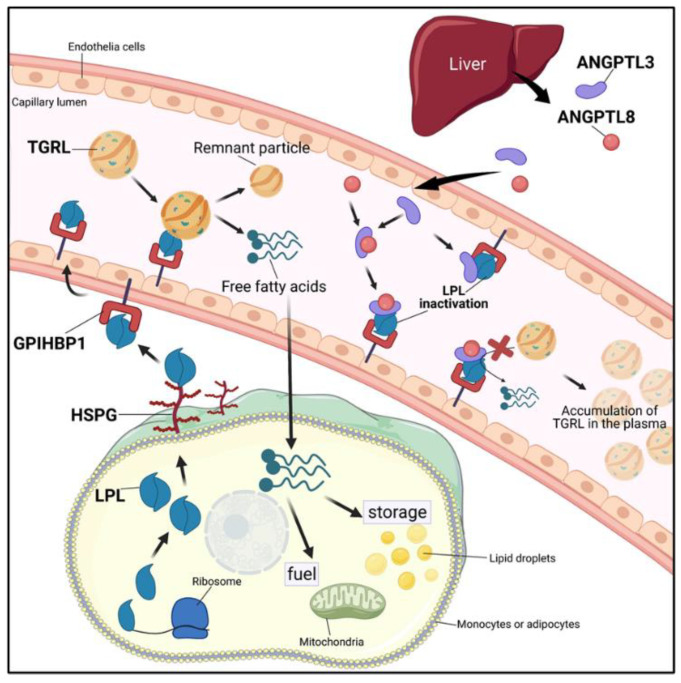
Overview of the role of ANGPTL3 in LPL inhibition and TGRL metabolism. Dietary lipid is transported via the blood as part of TG-rich lipoproteins (TGRLs) such as chylomicrons and VLDL, and the TGs are hydrolyzed by lipoprotein lipase (LPL). LPL is synthesized by myocytes or adipocytes and transferred to the cell surface by heparan sulfate proteoglycans (HSPGs). LPL is further transported to the glycosylphosphatidylinositol-anchored high-density lipoprotein binding protein 1 (GPIHBP1) upon the capillary endothelium. ANGPTL3 and ANGPTL8 proteins are produced and secreted by the liver. The ANGPTL3 protein alone or forming a functional complex with ANGPTL8 could markedly inhibit LPL activity (created with BioRender.com, accessed on 2 July 2021).

The lipoprotein lipase family consists of pancreatic lipase (PL), LPL, hepatic lipase (HL), and EL. The lipolytic processing of TGRLs by LPL plays a critical role in transportation and metabolism, providing lipids for fuel or storage. The free fatty acids released from TGRLs are taken up to generate energy by peripheral tissues such as heart and muscles or fat storage by adipose tissue [37,38]. Figure 3 shows the role of ANGPTL3 in TGRLs metabolism. LPL is produced and secreted by myocytes and adipocytes and then attaches to the cell surface through heparan sulfate proteoglycans (HSPGs) [39]. The protein glycosylphosphatidylinositol-anchored high-density lipoprotein binding protein 1 (GPIHBP1) is responsible for the transportation of LPL from the cell surface to the endothelium and flipping into the lumen of capillaries [40,41]. ANGPTL3 functions as an inhibitor of LPL to suppress the hydrolysis of the TG portion in TGRLs on the capillary endothelium. The function of ANGPTL3 is enhanced by ANGPTL8 [16]. Studies have shown that the ANGPTL3 overexpression-mediated increase in plasma TG levels could be attenuated in the absence of ANGPTL8. During feeding, the action of ANGPTL3 is driven by ANGPTL8, which forms a functional ANGPTL3/8 complex to enhance the ANGPTL3-mediated inhibitory effect of LPL activity [10,42,43]. Recently, Jin et al. reported that ANGPTL3/8 interacts with LPL and promotes furin-mediated LPL cleavage [44]. More insight into the roles of the ANGPTL3/8 complex and its molecular mechanism of action in LPL inhibition should be provided in molecular modeling studies in the future.

ANGPTL3 also exerts its action in adipose tissue and muscles. In adipose tissue, ANGPTL3 activates lipolysis, increasing the release of free fatty acids and glycerol from adipocytes. Wang et al. reported that ANGPTL3 plays a critical role in promoting the uptake of VLDL into white adipose tissue (WAT) and regulating energy homeostasis in the feeding state [45]. In *Angptl3*^-/-^ mice, the uptake of glucose into WAT and insulin sensitivity are elevated, resulting in conversion to fatty acids in WAT [45]. ANGPTL3 also inhibits LPL activity in muscles and elevates TGRL levels in circulation. In the feeding state, ANGPTL8 is induced, and the ANGPTL3/8 pathway is activated to inhibit LPL in cardiac and skeletal muscles, resulting in TG absorption by WAT [46]. These findings reveal the important role of ANGPTL3 in regulating LPL activity for energy substrate storage and utilization in WAT and muscle.

### 3.2. The Genetics of ANGPTL3 in Plasma Lipids

Several genetic studies support the critical role of ANGPTL3 in the regulation of plasma TGs in humans. By genome-wide association studies (GWAS), three single-nucleotide polymorphisms (SNPs), rs12130333, rs1748195, and rs2131925, at specific chromosomal loci near the *ANGPTL3* gene were linked to TG levels [47,48]. *ANGPTL3* rs12130333 is also markedly associated with Fredrickson hyperlipoproteinemia type 5, which is characterized by hyperlipidemia with high levels of chylomicrons and VLDL in plasma [49]. By exon sequencing analysis, a multiethnic sample of 3551 individuals from the Dallas Heart Study identified multiple loss-of-function nonsynonymous (NS) mutations in the *ANGPTL3* gene that were associated with low TG levels in plasma but not with other metabolic phenotypes. Functional studies of these NS variants in HEK293A cells showed that several mutant alleles of *ANGPTL3* strongly attenuate the secretion of the protein or its activity for inhibiting LPL [50]. Another study by exome sequencing of 15,994 genes from two individuals of a family with hypobetalipoproteinemia identified two novel compound heterozygotes carrying the loss-of-function mutations S17X and E129X in the first exon of *ANGPTL3* [51]. There are almost no ANGPTL3 proteins, and very low plasma LDL-C and TG levels can be detected in these two carriers. The S17X mutation in *ANGPTL3* resulting in familial hypobetalipoproteinemia was first reported in an Italian family in 1991 [52]. Individuals homozygous for S17X have almost no detectable ANGPTL3, notably higher LPL activity, and an obvious reduction in lipid levels in their plasma. The *ANGPTL3* S17X mutation is not associated with any changes in hepatic and endothelial lipase activities [53,54,55]. In the DiscovEHR human genetics study by exon sequencing of *ANGPTL3* in 58,335 participants, individuals carrying heterozygous loss-of-function mutations in *ANGPTL3* had markedly lower serum levels of TGs, HDL-C, and LDL-C than participants without these mutations [56]. Several studies have also demonstrated that homozygous or compound heterozygous variants encompassing nonsense, missense, splicing, and frameshift mutations in *ANGPTL3* gene cause familial combined hypolipidemia [57,58,59,60,61]. Notably, no evidence was found of an association between the risk of liver abnormalities or CVD and familial combined hypolipidemia [53].

In addition to lowering TGs, *ANGPTL3* homozygous variants were found to strongly reduce the levels of plasma total cholesterol, LDL-C and HDL-C [62]. In a meta-analysis of 21,980 patients with CVD and 158,200 control individuals, the results showed that total cholesterol and LDL-C levels were notably lower in carriers with loss-of-function mutations in *ANGPTL3*; however, HDL-C was not significantly different between carriers and noncarriers [58]. These data are consistent with the results from a GWAS, which found no association between mutations in the *ANGPTL3* gene and plasma HDL-C concentration, suggesting that ANGPTL3 may not be involved in the regulation of plasma HDL-C levels in humans. These findings suggest that mutations in *ANGPTL3* differentially affect lipid metabolism. It is possible that the diverse effects of ANGPTL3 on the different lipoproteins and cholesterol levels may be conferred by distinct functional domains in the ANGPTL3 protein. In addition to affecting the levels of plasma TGs and cholesterol, low NEFA levels were found in individuals with loss-of-function mutations in *ANGPTL3* in a mouse study, suggesting that ANGPTL3 could regulate plasma NEFA levels and lipolysis in adipose tissue [54]. These genetic data strongly link the antagonism of ANGPTL3 in humans to reduced levels of plasma lipids and decreased risk of CVD.

### 3.3. ANGPTL3 and Atherosclerotic Cardiovascular Disorders (ASCVDs)

Emerging evidence reveals that elevated plasma LDL-C and TG levels play essential roles in the development of ASCVDs. Modulation of LDL-C and TG levels may effectively control the progression of ASCVDs [58,63]. Individuals carrying loss-of-function mutations of *ANGPTL3* or mice with *ANGPTL3* deficiency had decreased plasma levels of LDL-C, TGs, and TGRLs, suggesting that downregulation of ANGPTL3 expression may lead to a low risk of ASCVDs in humans. ANGPTL3 deficiency was found to be associated with lower odds of ASCVDs. In an analysis of 13,102 individuals with CVDs, carriers with loss-of-function mutations in *ANGPTL3* had an approximately 40% lower risk of CVDs than noncarriers in humans [56]. In a mouse study, the authors also further found that treatment with a monoclonal antibody, evinacumab, for antagonism of ANGPTL3 was associated with a significant reduction in plasma TG and cholesterol levels as well as atherosclerotic plaques in the aortic root [56]. These data reveal a correlation between ANGPTL3 and the risk of ASCVDs. In a cohort-based meta-analysis study, a 34% reduced risk of CVDs was found among carriers of an *ANGPTL3* loss-of-function mutation compared with noncarriers, indicating that ANGPTL3 might influence the progression of CVDs [58].

## 4. Pharmacological Inactivation of ANGPTL3

**Figure 4:**
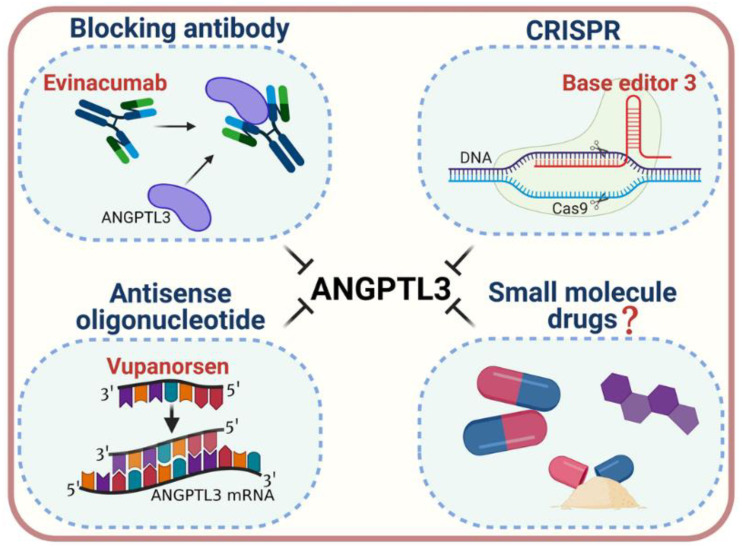
Strategies for pharmacological inactivation of ANGPTL3. Various strategies have been developed to pharmacologically inactivate ANGPTL3, which include blocking monoclonal antibodies (such as Evinacumab), antisense oligonucleotides (such as Vupanorsen), and a CRISPR genome editing system (such as base editor 3, BE3) for treatment of dyslipidemia. The development of oral, small-molecule inhibitors may serve as a novel pharmacological approach for ANGPTL3 inactivation (created with BioRender.com, accessed on 2 July 2021).

The notable effect of ANGPTL3 on lipid metabolism has sparked interest in ANGPTL3 as a molecular target for the prevention or treatment of dyslipidemia and ASCVDs. To date, three strategies for inactivation of ANGPTL3, antisense oligonucleotides (ASOs) [64], a monoclonal antibody (evinacumab) [56,65], and CRISPR-Cas9 genome editing technology [66,67,68], have been proposed (Figure 4). ANGPTL3 ASOs and evinacumab could decrease lipid levels both in animal models and in clinical trials in patients affected by homozygous familial hypercholesterolemia. In vivo genome editing by the CRISPR-Cas9 approach (namely, Base Editor 3, BE3) is based on an innovative mechanism to introduce mutations in *ANGPTL3* and has been demonstrated to reduce lipid levels in mice. The efficacy and safety of this therapeutic strategy remain unclear and need to be investigated in further studies [69].

### 4.1. ANGPTL3 ASOs as a Lipid-Lowering Therapy

ASOs targeting ANGPTL3 mRNA have been developed as inhibitors and have been studied in mice and humans [64,70]. In animal studies, ANGPTL3 ASOs showed limited efficacy in normocholesterolemic mice, whereas in hypercholesterolemic models with different lipid backgrounds, including *Ldlr*^−/−^, *ApoC3*^−/−^ *Ldlr*^−/−^ and *ApoC3*^+^ *Ldlr*^−/−^ mice, ANGPTL3 ASOs effectively decreased the levels of hepatic ANGPTL3 mRNA and plasma ANGPTL3 protein, resulting in marked reduction of plasma TGs, LDL-C and, to a lesser extent, HDL-C levels. In addition, ANGPTL3 ASOs have found to reduce atherosclerotic progression in *Ldlr*^−/−^ mice. ANGPTL3 ASOs reduced hepatic steatosis and improved insulin tolerance in diet-induced obese mice [64]. In a phase I clinical trial, the effect of ANGPTL3 ASOs was examined in 44 healthy male and female adults (age 18–65 years). A single subcutaneous injection of ANGPTL3 ASOs (80 mg) effectively reduced ANGPTL3, TGs, VLDL, non-HDL-C, and total cholesterol in plasma. Multiple-dose injection of ANGPTL3 ASOs (0–60 mg/week) for 6 weeks resulted in a dose-dependent reduction in plasma levels of ANGPTL3, TGs, non-HDL-C, and APOC3, which returned to normal after cessation of ASOs administration [64]. These studies reveal that ANGPTL3 ASOs effectively ameliorate plasma lipoproteins and atherosclerosis in mice and humans.

### 4.2. ANGPTL3 Monoclonal Antibody as a Lipid-Lowering Therapy

Evinacumab, a fully human monoclonal antibody that specifically binds to ANGPTL3, has been developed and proven to reverse ANGPTL3-mediated inhibition of LPL activity in vitro and in vivo [71]. In preclinical animal studies, evinacumab increased post-heparin LPL activity and reduced plasma levels of TGs, LDL-C and HDL-C in normolipidemic and hyperlipidemic mice. Similar effects on the plasma lipid profile were also found in cynomolgus monkeys with dyslipidemia, except that the LDL-C levels were unchanged [71]. In the hypercholesterolemic APOE*3Leiden CETP mice, evinacumab markedly reduced atherosclerotic lesions, concomitant with a significant reduction in plasma levels of TGs, VLDL and total cholesterol [56].

In a phase I clinical trial in human volunteers administered both intravenous and subcutaneous injection of evinacumab, researchers found that evinacumab-mediated inhibition of ANGPTL3 was associated with a dose-dependent reduction in plasma levels of TGs [56]. Evinacumab also reduced plasma LDL-C (−23.2%) and HDL-C (−18.4%) levels, although the decreases in these lipoproteins were much less pronounced than those in TGs (−76%) [56]. Based on data from two phase I studies, Ahmad et al. reported that evinacumab could reduce TGs and other lipids by ANGPTL3 inhibition in healthy human volunteers and in the hypertriglyceridemic subjects [72]. In a phase II trial of ANGPTL3 inhibition involving 9 patients with homozygous familial hypercholesterolemia, evinacumab treatment resulted in a mean reduction from baseline of 49% in the LDL-C level. In a phase III clinical trial, researchers randomly assigned 65 patients with homozygous familial hypercholesterolemia who were treated with a maximum dose of lipid-lowering medications to receive an intravenous injection of evinacumab (15 mg/kg) or placebo every 4 weeks. The outcome at 24 weeks of treatment showed decreases in plasma LDL-C levels from baseline of 49% in the evinacumab group [73]. These clinical studies indicate that pharmacological inactivation of ANGPTL3 shows efficacy and several potential benefits in patients with dyslipidemia.

## 5. Conclusions

Genetic, functional, and pharmacological studies have led to the conclusion that antagonizing or inhibiting ANGPTL3 could effectively enhance LPL and EL activity, modulate TG-rich lipoproteins and lipid metabolism, and reduce the risk of cardiovascular events. However, several questions regarding the role and regulation of ANGPTL3 in lipid metabolism remain to be clarified. How can inhibition of ANGPTL3 lead to reduced plasma LDL-C levels? One possibility is that inhibition of ANGPTL3 promotes VLDL clearance or decreases VLDL secretion [24], thereby reducing the conversion of VLDL to LDL, resulting in decreased LDL-C levels. This assumption needs to be verified. How can ANGPTL3 affect lipolysis in adipose tissue and the release of fatty acids? The molecular mechanisms and potential receptors of ANGPTL3 involved in adipose tissue lipolysis need to be investigated to clarify this point. In addition to the TG-lowering effect, the outcome of clinical trials is related to the role of ANGPTL3 in controlling plasma levels of LDL-C and total cholesterol. Traditional lipid-lowering therapies such as statins and PCSK9 monoclonal antibodies are dependent on LDL receptor (LDLR) expression. The cholesterol-lowering effect of ANGPTL3 has raised interest in ANGPTL3 as a target for the treatment of patients with hypercholesterolemia that show little response to traditional therapies or have no LDLR activity. To date, the absence of oral and affordable low molecular weight ANGPTL3 inhibitors has limited the beneficial effect of these new medications. The epitope of ANGPTL3 targeted by evinacumab remains unclear. Understanding the structure of the binding region of this monoclonal antibody by molecular modeling analysis may help to develop small-molecule inhibitors of ANGPTL3. The rational design of oral, small molecules as ANGPTL3 inhibitors may serve as a novel pharmacological approach for the treatment of dyslipidemia.

## Data Availability

Not applicable.
